# Meperidine compared to morphine for rigors associated with monoclonal antibody-related infusion reactions

**DOI:** 10.1177/10781552241259986

**Published:** 2024-06-17

**Authors:** Hanna Yakubi, Aaron Paul Steele, Megan Tsao

**Affiliations:** 1 Department of Pharmacy, 70083 University of California at Davis Health System, Sacramento, CA, USA

**Keywords:** Monoclonal antibody, rigors, infusion reaction, meperidine, morphine

## Abstract

**Introduction:**

Infusion reactions, characterized by symptoms such as rigors, fever, and hypotension, are common adverse events that occur during monoclonal antibody (MAB) therapy. The treatment of rigors often involves opioids, most commonly meperidine, despite limited evidence supporting use in the setting of MAB infusions. This study aims to compare the efficacy and safety of intravenous (IV) meperidine and morphine is treatment of MAB-related rigors, filling a significant gap in the literature.

**Methods:**

This was a single-center, retrospective cohort study which reviewed patients either inpatient or within outpatient infusion centers from January 2015 to January 2024. Patients receiving IV 2 mg morphine or 25 mg meperidine for MAB-related rigors were included. The primary outcome was defined as the number of opioid doses required for rigors ablation. Secondary outcomes included rates of naloxone administration and documented sedation.

**Results:**

A total of 1251 administration events were screened, of which 127 and 26 rigor events were in the meperidine and morphine cohorts, respectively, were included. A majority of both cohorts required only one dose of either agent for rigors ablation with <20% of either cohort requiring 2 or more doses (p = 0.539). Low rates of sedation were observed in both groups.

**Conclusion:**

Both meperidine and morphine effectively manage MAB-related rigors within minimal safety concerns. These findings suggest that morphine is a suitable alternative to meperidine for this indication, which may influence future formulary decision, provide alternatives for drug shortage, and optimize supportive care for patients undergoing MAB therapy.

## Background

Infusion reactions are a common adverse event to monoclonal antibody (MAB) intravenous infusions. Symptoms may include rigors, fever, hypotension, anaphylactic shock, among others.^1^ Infusion reactions, including the rigors component, can be a distressing experience for patients. The biochemical pathway of rigors, both in the setting of infusion reactions and other clinical situations, is complex and includes an amalgam of receptors, such as opioid, adrenergic, and cholinergic.^2^ At many institutions, intravenous opioids are the treatment of choice for shivering or rigors.

Despite widespread use of opioids for rigors, evidence supporting this indication is limited. Meperidine is often preferred due to its unique binding of both mu and kappa opioid receptors, unlike morphine or fentanyl which primarily targets mu receptors.^3^ This mechanism may enhance rigors mitigation with meperidine treatment. Comparative data on meperidine and other opioids, or alternative treatments, are limited to postanesthesia shivering. A single-center, double-blinded randomized trial in adult patients experiencing shivering after general anesthesia found similar response rates at 5 min with meperidine and clonidine and overall poor response with urapidil (90% vs 80% vs 30%, respectively). Furthermore, an analysis of various opioids for postanesthesia shivering in adults found faster resolution of shivering with fentanyl and meperidine compared to morphine, although shivering recurrence was similar between agents.^4^ To date, an investigation by Lucas and colleagues is the sole published data assessing the efficacy of any opioid—morphine, in this instance—for the termination of rigors caused by monoclonal antibodies and reported a 97% efficacy rate.^5^ Overall, there is limited comparative literature to guide the selection and optimization of rigors treatment, particularly those secondary to MAB infusions.

At University of California (UC) Davis Health, meperidine was removed from formulary in January 2023 due to concerns for reported over-sedation when used for other indications. Subsequently, morphine has been adopted as the opioid of choice for MAB-related rigors and is included in pertinent treatment protocols. This study aims to present evidence comparing the safety and efficacy of meperidine compared to morphine for the treatment of MAB-related rigors, addressing a significant gap in current medical literature.

## Methods

This study was designed as a single-center, retrospective, nonrandomized cohort study at the University of California, Davis Health System from January 1, 2015 to January 1, 2024. The study was conducted in accordance with the Declaration of Helsinki and was reviewed by the Institutional Review Board of UC Davis (no. 2086387-1, expiration date: April 14, 2025) on September 8, 2023, with the need for written informed consent waived. Patient data was de-identified and will not be shared with third parties.

Due to formulary withdrawal of meperidine and subsequent substitution with morphine, patients within the meperidine group were assessed from January 1, 2015 to December 31, 2022 and patients within the morphine group were assessed from January 1, 2023 to January 1, 2024. Adult patients were included if they received one or more doses of intravenous (IV) morphine or IV meperidine for the indication of rigors secondary to MAB infusion reactions. Patients were excluded if morphine or meperidine were used for indications other than rigors or for rigors due to non-MAB medications, such as amphotericin. Patients treated both inpatient and at outpatient infusion centers were included.

The primary outcome was defined as the number of morphine or meperidine doses required for rigors ablation. Institutional order sets for MAB-related rigors allow for 2 doses of either as needed morphine 2 mg IV or meperidine 25 mg IV given at least 20 min apart, at which point additional orders are required for further doses. Secondary outcomes to assess safety include rates of naloxone administration and nursing or physician documentation of “sedation” within the electronic medical system in the immediate 24 hours after administration of IV opioid. Descriptive statistics were used to assess baseline and demographic data. Where appropriate, adverse events were graded using the Common Terminology Criteria for Adverse Events, version 5. Continuous data was assessed using a standard student t-test. Categorical data was assessed using chi-square or fisher's exact test. A p-value of ≤ 0.05 was considered statically significant. Statistical analysis was performed using GraphPad® and Microsoft Excel®.

## Results

A total of 1251 morphine or meperidine administration events were screened, of which 127 and 26 rigor events met inclusion criteria for the meperidine and morphine cohorts, respectively. Of these events, 96 (63%) occurred within an inpatient unit during hospital admission while 57 (37%) occurred within the outpatient infusion center. The total patient population includes 125 unique patients, 100 among the meperidine group and 25 among the morphine group. The most common reason for exclusion was administration for pain (34%), followed by patient age (28%) and administration for post procedure shivering (19%). Baseline patient and treatment characteristics are presented in Table 1. Baseline characteristics were well balanced between the treatment groups, including similar mean age (62 vs 64 years, p = 0.4414) and prior history of opioid allergy (9% vs 12%, p = 0.7741). Although there were numerically a higher proportion of patients with reduced renal function, defined as creatinine clearance <50 mL/min, among morphine patients, this difference was not statistically significant (18% vs 35%, p = 0.0594). Hematologic malignancies were predominant in both cohorts. Monoclonal antibodies targeting the CD20 isotope were the most common causative agent in both groups. The majority of infusion reactions were grade 2 and occurred within the first 2 doses of MAB.

**Table 1.** Patient and treatment characteristics.

Characteristic	Meperidine (n = 127)	Morphine (n = 26)	p-Value
Age, mean (SD)	62 (17)	64 (13)	0.4414
Sex, female	32 (25)	9 (35)	0.0011
History of opioid allergy	12 (9)	3 (12)	0.7741
Diagnosis			0.9944
NHL	71 (56)	13 (50)	
CLL	26 (20)	6 (23)	
ALL	18 (14)	5 (19)	
Other oncology*	7 (6)	0	
Other nononcology**	5 (4)	2 (8)	
CrCl < 50 mL/min	23 (18)	9 (35)	0.0594
Monoclonal antibody			0.9878
Rituximab	106 (83)	19 (73)	
Obinutuzumab	15 (12)	7 (27)	
Daratumumab	4 (3)	0	
Ocrelizumab	1 (1)	0	
Trastuzumab	1 (1)	0	
Reaction within first 2 doses of MAB	90 (71)	22 (85)	0.1492
Infusion reaction grade^†^			0.9580
2	119 (94)	25 (96)	
3	5 (4)	1 (4)	
4	3 (2)	0	
Diphenhydramine administered before or after MAB infusion ^§^	127 (100)	26 (100)	–

All figures presented n (%) unless otherwise noted.

*Meperidine cohort: four patients with multiple myeloma, two patients with hairy cell leukemia, one patient with breast cancer.

**Meperidine cohort: one patient each with autoimmune lymphoproliferative syndrome, autoimmune hemolytic anemia, rheumatoid arthritis, hemolytic uremic syndrome, and multiple sclerosis; morphine cohort: one patient each with multiple sclerosis and immune thrombocytopenia.

† Graded per Common Terminology Criteria for Adverse Events, version 5.

§ Given either as part of premedication or part of management of infusion reaction.

ALL: acute lymphoblastic leukemia; CLL: chronic lymphocytic leukemia; CrCl: creatinine clearance; MAB: monoclonal antibody; NHL: non-Hodgkin lymphoma; SD: standard deviation.

**Figure 1.**
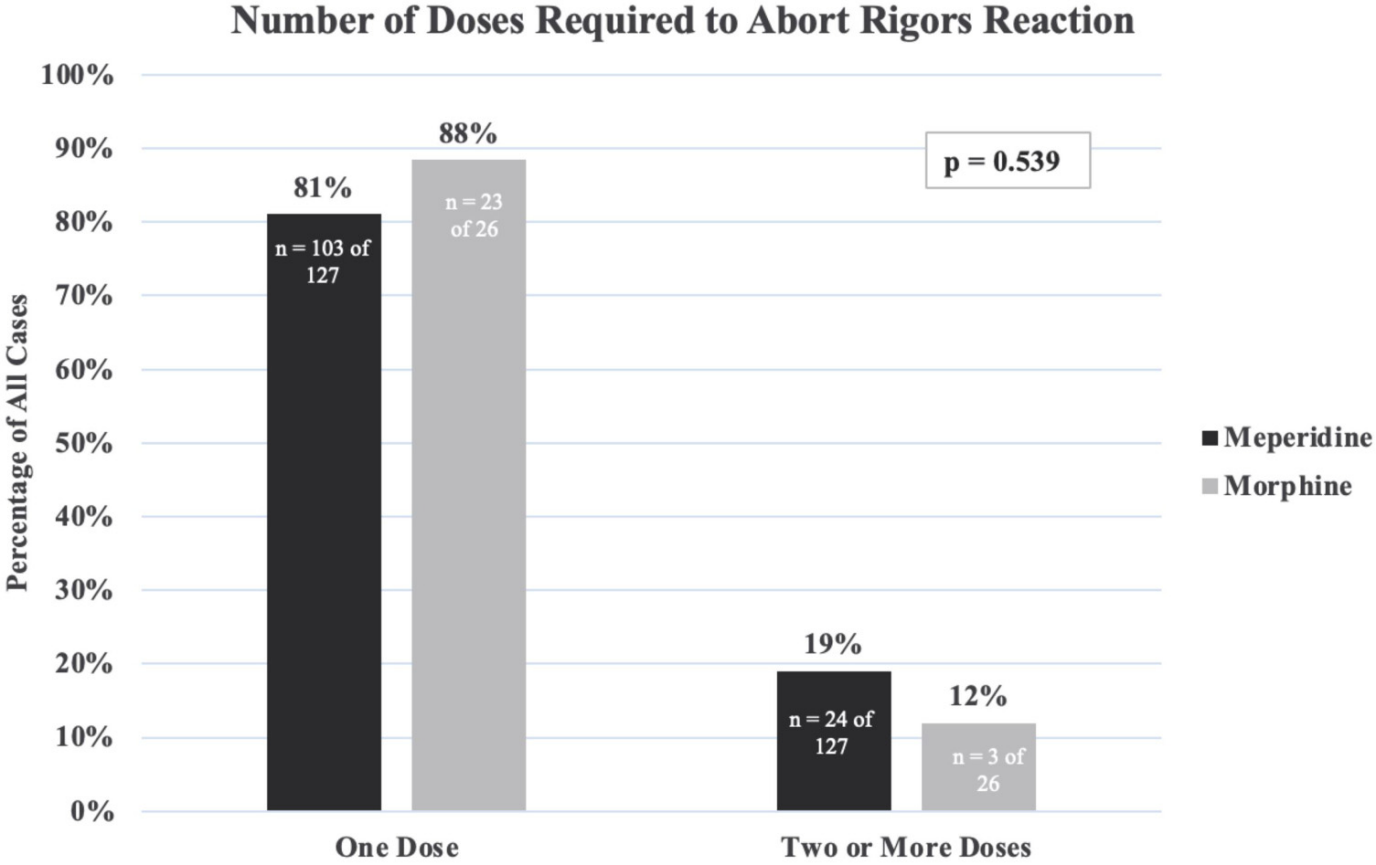
Number of doses required to abort rigors reaction.

Results of the primary outcome are presented in Figure 1. We observed a similar proportion of patients in both cohorts requiring escalation to two or more doses of intravenous opioid (19% among meperidine cohort vs 12% among morphine cohort), indicating that a single dose was sufficient to achieve rigors ablation in most patients (p = 0.539). A minority of patients required additional opioid doses beyond what is specified within the institutional order set. Specifically, in the meperidine cohort, 2% (2 patients) required 3 doses for rigors cessation and 4% (1 patient) in the morphine group required up to 6 doses. The case requiring six morphine doses involved a severe grade 3 infusion reaction during outpatient rituximab administration for diffuse large B-cell lymphoma, requiring hospital admission. Among patients in the meperidine group requiring three or more doses, we identified one case associated with a grade 3 reaction to obinutuzumab in the treatment of chronic lymphocytic leukemia. The other two cases, involving daratumumab and rituximab administration for multiple myeloma and primary central nervous system lymphoma, respectively, were associated with grade 2 infusion reactions. Of note, three of four (75%) instances where patients needed three or more opioid doses for rigors ablation occurred during the first dose of MAB therapy. Across the entire study cohort, there were three case of grade 4 infusion reaction requiring upgraded care to the intensive care unit. However, all three cases were successfully managed with only one dose of meperidine for rigors cessation.

**Figure 2.**
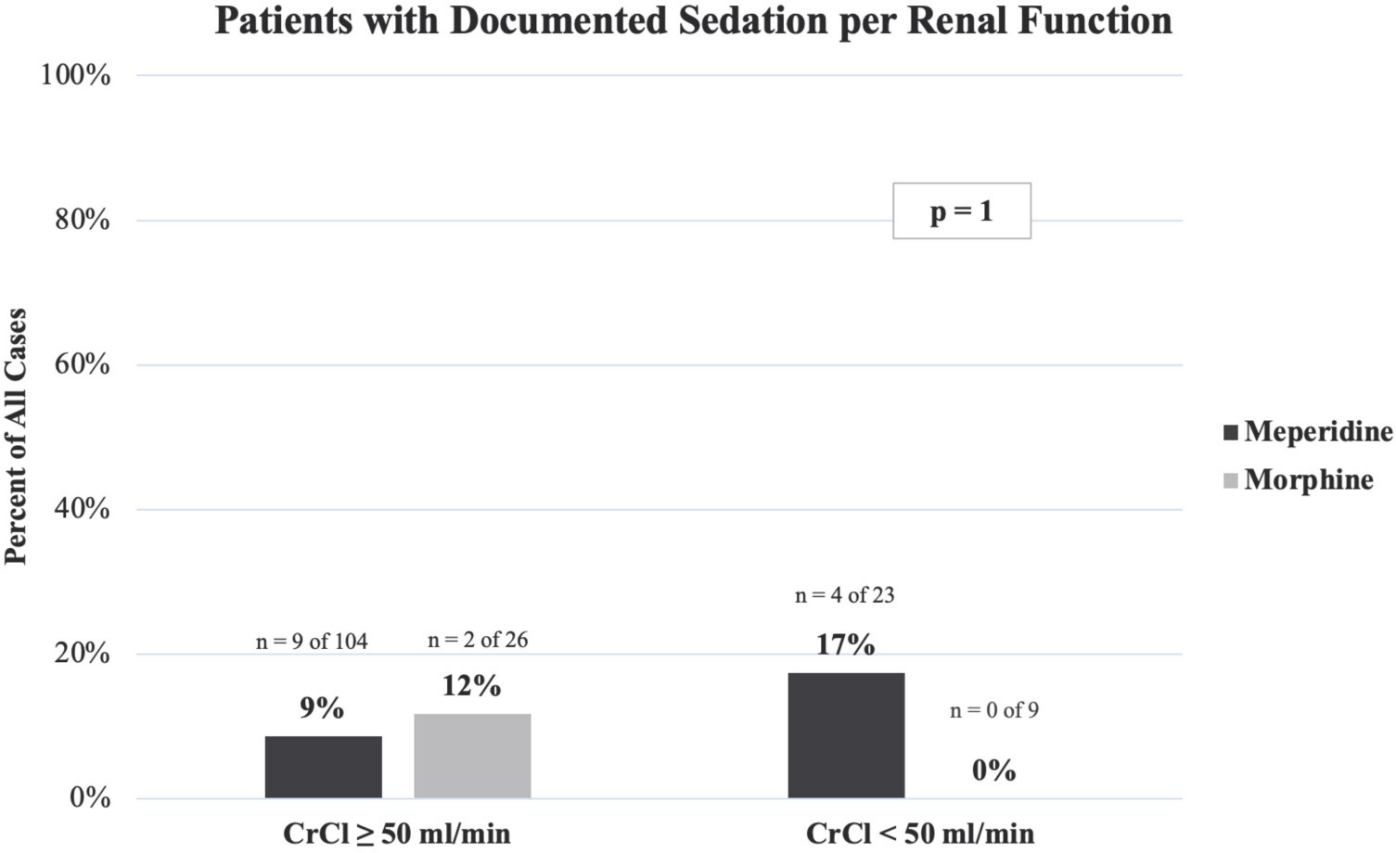
Patients with documented sedation per renal function.

There were no instances of naloxone administration in either cohort. Among the meperidine group, 10% of patients experienced a documented sedation event compared to 9% of morphine patients (p = 0.972). Documented sedation event rates designated per renal function are presented in Figure 2. In total, sedation was documented in 9 of 104 (9%) meperidine patients and 2 of 26 (12%) morphine patients with preserved renal function. Among patients with reduced renal function, 4 of 23 (17%) meperidine patients and no morphine patients experienced a documented sedation event. This difference was not statistically significant (p = 1).

## Discussion

This is the first study to compare different opioids for the treatment of MAB-related rigors. Although meperidine is a commonly used agent for this indication, this study shows that meperidine 25 mg IV and morphine 2 mg IV provided similar resolution of rigor symptoms among patients experiencing MAB infusion reactions. Low rates of sedation were seen with both agents and there were no initial safety signals to suggest patients with poor renal function are at higher risk of sedation. Most infusion reactions occurred among admitted patients which were an unexpected result as about 75% of monoclonal antibody doses are given in the outpatient setting at our institution. This finding is likely explained by known infusion reaction timing of common monoclonal antibodies, such as rituximab or obinutuzumab, in which reactions typically occur with the first dose when disease burden is highest.^1,6^ Patients are often admitted for first doses for monitoring, further workup, and other oncology protocol requirements.

The results of this study are in line with previous reports in the setting of postoperative shivering. Meperidine, either at flat doses of 25 to 50 mg or weight based dosing of 0.4 mg/kg, has been shown to be effective at shivering resolution in 89% to 90% of reported cases.^4,7,8^ Low rates of adverse events are noted both in previous meperidine literature and the results of the current study.^4,7,8^ Interestingly, data from postoperative shivering patients shows comparatively worse outcomes with morphine, both with lower response rates at about 40% and longer time to ablation by 2 to 3 min when compared to other opioids.^4,8^ On the other hand, the study by Lucas et al.^5^ showed high rates of efficacy and low rates of side effects when morphine was used to treat rituximab-induced rigors, results which are consistent with our findings. The incongruence in morphine outcomes between postoperative patients compared to MAB patients is unclear and may be related to different thermoregulatory pathways underlying the manifestation of rigors in these different patient populations.

There are limitations to this study. First, there are significantly more patients in the meperidine group compared to the morphine group which may not accurately reflect differences in adverse event incidences, particularly those that are believed to be rare such as sedation. Because of the differences in sample size, outcomes should be seen as trends that require long-term follow up, particularly in the renal subgroup. Furthermore, although naloxone administration represents an objective data point related to sedation, this event was, as expected, very rare. Thus, we relied on documented sedation within electronic medical record notes that may be influenced by differences in provider/nursing assessment and documentation practices. Moreover, outpatient MAB infusions may not capture late-onset sedation related to opioid administration and, thus, overall sedation rates may be underreported in this cohort. Also, the use of concomitant sedating medications, except diphenhydramine, was not assessed which may confound sedation outcomes. However, all patients in both groups received diphenhydramine as either premedication or for treatment of the infusion reaction, and thus the use of this agent specifically is balanced between groups. Prospective study design may rectify these issues in future studies. Next, this study was not designed to assess differences in time to rigors resolution which may further guide decision making when choosing the optimal opioid for this indication. Finally, the results of this study may not apply to MABs in which rigors may be secondary to a cytokine-related process, for which the utility of opioid therapy is unclear.

## Conclusion

Despite the limitations, the results of this study can play a critical role in optimizing supportive care for patients receiving MAB therapy and will provide additional data for evidence-based formulary decisions. Although many clinicians may be more comfortable with meperidine for this indication given the historical use, there can be various reasons why alternative therapy is warranted, including allergies, drug shortages, and concerns regarding the potential serotonergic and neurotoxic properties of meperidine.^9^ This study indicates that morphine is an appropriate alternative, both in response and safety. Future studies should focus on differences in time to resolution and reoccurrence of rigors following opioid use for MAB-related rigors. Also, comparing opioids to other agents that target alternative receptors, such as intravenous ondansetron or clonidine, may also shed light on potential opioid-sparing options for treatment of MAB-related rigors.

## Supplemental Material

sj-xlsx-1-opp-10.1177_10781552241259986 - Supplemental material for Meperidine compared to morphine for rigors associated with monoclonal antibody-related infusion reactions Supplemental material, sj-xlsx-1-opp-10.1177_10781552241259986 for Meperidine compared to morphine for rigors associated with monoclonal antibody-related infusion reactions by Hanna Yakubi, Aaron Paul Steele and Megan Tsao in Journal of Oncology Pharmacy Practice
